# Arterial lactate as a predictor of postoperative complications in head and neck squamous cell carcinoma

**DOI:** 10.1016/j.bjorl.2021.04.008

**Published:** 2021-05-06

**Authors:** Suzane Pereira de Souza, Maurício Gomes da Silva Serra, Neyara dos Santos Oliveira, Márcio Campos Oliveira, José de Bessa Junior, Tercio Guimarães Reis

**Affiliations:** a Universidade Estadual de Feira de Santana (UEFS), Colegiado de Medicina, Feira de Santana, BA, Brazil; b Hospital Geral Clériston Andrade, Feira de Santana, BA, Brazil; c Universidade Estadual de Feira de Santana (UEFS), Feira de Santana, BA, Brazil; d Universidade Estadual de Feira de Santana (UEFS), Departamento de Saúde, Feira de Santana, BA, Brazil; e Santa Casa de Misericórdia de Feira de Santana, Feira de Santana, BA, Brazil

**Keywords:** Lactate, Head and neck neoplasms, Postoperative complications

## Abstract

**Introduction:**

Surgery is one of the most frequently used options in the treatment of head and neck squamous cell carcinoma. In surgical patients, the use of arterial lactate to assess hypoxemia and severe inflammatory states is well-founded. However, there are few studies on its use in patients with head and neck squamous cell carcinoma. The aim of this study was to investigate whether the serum arterial lactate level on the 1st postoperative day would be a predictor of postoperative complications in head and neck squamous cell carcinoma surgeries.

**Methods:**

This is a prospective cohort, which evaluated 44 adult patients of both genders, with HNSCC, who underwent surgery associated with monobloc neck dissection as an initial treatment. Patients were divided into two groups, according to the presence or absence of postoperative complications: with complication (Clavien–Dindo II–V) and without complications (Clavien–Dindo 0–I). Student’s *t*-test and its variants were used to compare continuous data. Pearson’s or Spearman’s test was used to correlate the data and *p* values ​​<0.05 were considered statistically significant.

**Results:**

A total of 59% of the patients (n = 26/44) developed postoperative complications. Serum lactate was significantly higher in the group with complications when compared to patients without complications, respectively 2.15 mmoL/L (1.10–3.90) and 1.59 mmoL/L (0.70–3.44); *p* = 0.03. The prognostic accuracy of arterial lactate was 69% (95% CI: 54%–82%; *p* = 0.03), estimated by the ROC curve. A cut-off >1.7 mmoL/L was identified, with a sensitivity of 65.38% and specificity of 66.67%.

**Conclusion:**

Arterial lactate measured on the first postoperative day is a good predictor of postoperative complications in patients with head and neck squamous cell carcinoma.

## Highlights

• Arterial lactate is a good predictor of postoperative complications in head and neck surgery.
• An arterial lactate level > 1.7 mmoL/L increases the risk of postoperative complications in head and neck surgery.

## Introduction

Head and neck squamous cell carcinoma (HNSCC) typically appears in the oropharynx, oral cavity, hypopharynx and larynx.^1^ The estimate of the Brazilian National Cancer Institute (INCA, *Instituto Nacional do Câncer*) for the 2020−2020 period shows an estimated risk of 10.69 new cases of cancer of the oral cavity for every 100,000 men, occupying the fifth position. Laryngeal cancer, in the same period, will have an estimated risk of 6.20 new cases per 100,000 men and 1.06 new cases per 100,000 women in the national territory.^2^ Surgery and radiation therapy, with or without chemotherapy, have been the main treatment strategies for HNSCC.^3^ It is essential that surgical complications and the associated morbidity be minimized, with the approach used allowing maximum eradication of the disease, increasing survival and reducing hospital length of stay.^4^

Lactate is a product of glycolysis, obtained from pyruvate, particularly in the absence of oxygen. Traditionally, the increase in arterial lactate in hemodynamically unstable individuals is related to shock and/or arterial hypoxemia. Hyperlactatemia is a marker of the inflammatory status and screening for infections with an unknown focus in unexplained lactic acidosis is recommended.^5^ The advantages of using lactate include that the method is easily repeated and interpreted, of easy access and has a low operational cost.^6^

The decrease in serum lactate during the first 24 h indicates better outcomes than the persistence of hyperlactatemia.^5^ The course of blood lactate levels is useful for assessing the established therapy and it has been shown that the delay in lactate clearance is associated with poor outcomes in surgical patients.^7^ Patients who died after HNSCC surgery had elevated arterial lactate levels before surgery and in the first 24 postoperative hours.^6^ In a review^7^ that evaluated the role of the lactate level as a predictor of mortality and other complications in critically-ill patients, five publications on surgical patients were identified, of which only one was on the postoperative period of head and neck surgeries. There is a correlation in the literature between lactate increase and a worse prognosis in major surgeries, but there is little data regarding major head and neck surgeries.

The aim of this study was to investigate the role of serum arterial lactate measurement on the 1st postoperative day as a predictor of postoperative complications in HNSCC surgeries.

## Methods

This is a prospective cohort conducted by the Head and Neck Cancer Research Center (NUPESCAP, *Núcleo de Pesquisa em Câncer de Cabeça e Pescoço*), between November 2016 and September 2019, at Santa Casa de Misericordia hospital in the municipality of Feira de Santana, state of Bahia, Brazil.

The study design, data collection and use were carried out according to the provisions of Resolution N. 466/2012.^8^ The participants were invited by asking them to sign the Free and Informed Consent Form (FICF). The study was approved by the Research Ethics Committee under number 46664315.7.0000.0053.

The inclusion criteria were: patients over 18 years of age, of both genders, with cancer of the oral cavity and larynx, with clinical stages T1–T4,^9^ who underwent surgery associated with monobloc neck dissection as the initial treatment.

Patients under 18 years of age and those who chose to undergo nonsurgical treatment (radiotherapy and/or chemotherapy) were excluded from the study; also, those with diseases that altered the normal lactate metabolism, such as kidney and liver dysfunction; patients taking medications that could cause lactic acidosis, such as nucleoside analog reverse-transcriptase inhibitors (used to treat HIV/AIDS), linezolid, isoniazid and to a lesser extent, metformin and those who refused to sign the free and informed consent form (FICF).

A total of 44 patients who had arterial lactate levels measured within the first 24 postoperative hours were evaluated. The collected data included general information such as age, gender, level of schooling, income, primary diagnosis, life habits (alcohol consumption and smoking), clinical tumor staging, physical status classification according to ASA (American Society of Anesthesiology) to determine the risk of perioperative mortality and Charlson’s Comorbidity Index (CCI) collected preoperatively.

Arterial lactate was collected on the 1st postoperative day by arterial puncture and the samples were analyzed on a gasometer using the enzymatic method. In this technique, reagents determine the concentration of lactic acid by oxidizing the dinucleotide adenine nicotinamide (NAD) with the lactate, generating the pyruvate, NAD in oxidized form (NADH) and hydrogen ions.^10^

The complications were studied according to the Clavien–Dindo scale^11^ (Table 1). The patients were divided into two groups, according to the presence or absence of postoperative complications: patients with complications, that is, Clavien–Dindo II–V and those without postoperative complications, Clavien–Dindo 0–I.

**Table 1:** Clavien–Dindo grading of surgical complications.

Grade	Definition	
Grade I	Any deviation from the normal postoperative course without the need for pharmacological treatment or surgical, endoscopic, and radiological interventions.	
	Allowed therapeutic regimens are drugs as antiemetics, antipyretics, analgesics, diuretics, electrolytes, and physiotherapy. This grade also includes wound infections opened at the bedside.	
Grade II	Requiring pharmacological treatment with drugs other than such allowed for grade I complications.	
	Blood transfusions and total parenteral nutrition are also included.	
Grade III	Requiring surgical, endoscopic or radiological intervention	III a. Intervention not under general anesthesia.
		III b. Intervention under general anesthesia.
Grade IV	Life-threatening complication (including CNS complications)^a^	IV a. Single organ dysfunction (including dialysis)
	Requiring IC/ICU management.	IV b. Multiorgan dysfunction
Grade V	Death of a patient	
Disability	If the patient suffers from a complication at the time of discharge	
	(for “disability”), it is added to the respective grade of complication. This label indicates the need for a follow-up to fully evaluate the complication.	

CNS, Central Nervous System; IC, Intermediate Care; ICU, Intensive Care Unit.

a Brain hemorrhage, ischemic stroke, subarachnoid bleeding, but excluding transient ischemic attacks.

The quantitative variables were described by measures of central tendency (means or medians) and the respective measures of dispersion (standard deviation or interquartile range). The qualitative or categorical variables were described by their absolute values ​​or proportions. When comparing the continuous data, the Student's *t* test and its variants were used. The ROC curve was employed to evaluate the accuracy of the lactate levels and to calculate the value with the best discrimination property. Values of *p* < 0.05 were considered statistically significant. The software Graphpad Prism v. 8.03 for Windows, GraphPad Software, Inc. La Jolla, CA, USA, was used for the analysis of data and creation of the graphs.

## Results

Forty-four subjects with HNSCC undergoing surgery as the initial treatment, with a median age of 67 years (41–79), whose gender distribution showed a male predominance of 81.82% (n = 36/44) were analyzed. The sociodemographic and clinical characteristics of the population are detailed in Table 2.

**Table 2:** Sociodemographic and clinical characteristics of the participants.

Variables	Description	Values n (%)
Gender	Female	8 (18.18)
	Male	36 (81.82)
Level of schooling	Illiterate	9 (20.45)
	Incomplete Elementary School	29 (65.91)
	Complete Elementary School	1 (2.28)
	Incomplete High School	5 (11.36)
Income	Unemployed (no income)	9 (20.45)
	<1 minimum wage	25 (56.82)
	1–2 minimum wages^a^	10 (22.73)
Alcohol consumption	Yes	39 (88.64)
	No	5 (11.36)
Smoking	Yes	41 (93.18)
	No	3 (6.82)
Primary site	Oral cavity	21 (47.73)
	Larynx	23 (52.27)
Stage	I	11 (25.00)
	II	12 (27.27)
	III	20 (45.45)
	IVA	1 (2.28)
ASA	2	44 (100)
Charlson’s comorbidity index	0	27 (61.36)
	1	16 (36.36)
	2	1 (2.28)

a Minimum wage value at the time of the interview: R$ 880.00 (2016), R$ 937.00 (2017), R$ 954.00 (2018) and R$ 998.00 (2019).

Twenty-six (59%) patients (n = 26/44) developed postoperative complications. The serum lactate level was significantly higher in this group when compared to those who did not develop any complications, respectively 2.15 (1.10–3.90) mmoL/L and 1.59 (0.70–3.44) mmoL/L (*p* =  0.03).

**Figure 1:**
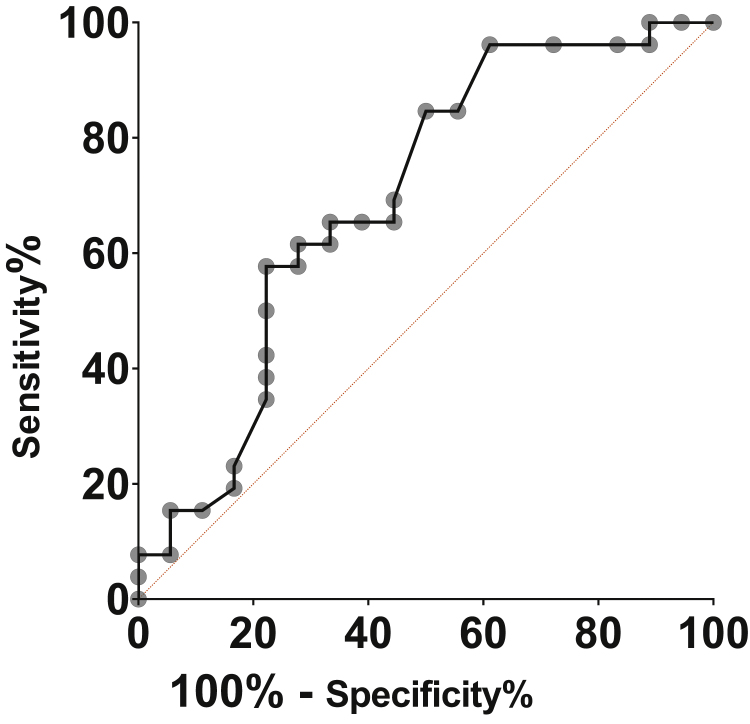
Lactate ROC curve on the 1st postoperative day in patients with HNSCC.

The prognostic accuracy of arterial lactate on the first postoperative day estimated by the ROC curve was 69% (95% CI: 54%-82%), with *p* =  0.03. Arterial lactate >1.7 mmoL/L was the best cutoff point, with a sensitivity of 65.38% and specificity of 66.67%. The accuracy measures for the different lactate values ​​are shown in Table 3 and Fig. 1.

**Table 3:** Measurements of lactate accuracy on the 1st postoperative day in patients with HNSCC.

Lactate	Sensitivity	Specificity	LR+^a^
>1.5	84.6	42.1	1.46
>1.6	69.2	57.9	1.64
>1.7	65.4	68.4	2.07
>1.9	57.7	68.4	1.83
>2.0	57.7	73.7	2.19
>2.1	50.0	73.7	1.90
>2.4	34.6	78.9	1.64
>2.7	23.1	84.2	1.46

a Index of probability.

## Discussion

The lactate level on the first postoperative day proved to be a good predictor of complications following the surgical treatment of HNSCC, with a prognostic accuracy of 69%.

INCA estimates that, together, the sites corresponding to the HNSCC occupy the second position regarding cancer prevalence in men.^2^ In India, head and neck cancers are the most prevalent in the population^12^ and in the United Kingdom they occupy the sixth place.^13^ In this cohort, regarding the primary site, 47.73% (n = 21/44) of the individuals were diagnosed with oral cavity squamous cell carcinoma (SCC) and 52.27% (n = 23/44) with laryngeal SCC. In the present study, the gender distribution showed a male predominance of 81.82% (n = 36/44), while the female gender represented 18.18% (n = 8/44). A French study on the profile of patients with HNSCC showed a similar distribution.^14^

In Eastern Europe, southern India and Latin America, the high incidence of these cancers in men is related to a combination of factors such as tobacco use, alcohol intake and dietary habits.^15^ Alcohol consumption and tobacco use are the risk factors most frequently associated with the disease.^1^, ^16^, ^17^ Alcohol intake was reported by 88.64% (n = 39/44) of the participants, while 93.18% (n = 41/44) had a history of smoking in the present study. In India, low socioeconomic levels and viral infections are also mentioned.^12^

As for the socioeconomic profile, the analysis of the cohort showed that 20.45% (n = 9/44) were unemployed; 56.81% (n = 25/44) had an income below one minimum wage and 22.72% (n = 10/44) had an income between one and two minimum wages. There was a predominance of individuals who had not finished elementary school; 65.91% (n = 29/44), the illiterate corresponded to 20.45% (n = 9/44); 2.28% (n = 1/44) had finished elementary school and 11.36% (n = 5/44) had not finished high school. A Scottish study found that high levels of education are protective against head and neck cancer, but this effect was lost after adjustments for tobacco smoking and alcohol consumption.^18^

Regarding the clinical staging, a representative part of the sample had an advanced clinical stage, with 45.45% (n = 20/44) in stage III and 2.28% (n = 1/44) in stage IVA, with another part in the initial stage, with 25% of the cases (n = 11/44) in stage I and 27.27% (n = 12/44) in stage II. A Scottish study showed all patients in stage III or IV and 60% already had metastasis in the cervical lymph nodes.^13^ In a region with a high incidence of the disease in France, 56.6% of the studied patients were already diagnosed in stage IV, with advanced disease being related to swelling in the neck, poor general health status and dyspnea.^14^

All patients in the present study were classified as ASA2. A Dutch study identified 53% of participants with a similar score.^19^ The Charlson Comorbidity Index (CCI) estimates the prognostic impact that some pre-existing clinical conditions have on mortality in one year, with a maximum of 6 points, with the score being proportional to the prognosis.^20^ In the present cohort, in relation to the CCI, 61.36% (n = 27/44) did not score; 36.36% (n = 16/44) attained one point and two points were attributed to 2.28% (n = 1/44). Guizard et al.^14^, using the CCI, verified that 11.7% of the patients did not score, while 47.5% scored one or two points, with 40.8% having a score greater than or equal to three. The fact that this study showed a considerable number of patients with advanced disease in its sample, (51.2% in stage IV)^14^ may have contributed to a worse CCI in relation to the studied population.

Ibrahim and Ahmed,^6^ in a prospective study of 322 patients with head and neck cancer undergoing major surgery in Cairo (Egypt), identified elevated lactate levels at admission and within 16 h postoperatively, in addition to an increased APACHE score and older age as predictors of complications. The median age in the present study was 67 (41–79) years, not very different from the mean age identified by the Egyptians, of 64.4 (44–84) years. In the postoperative evaluation of cardiac surgeries, lactate levels >1.5 mmoL/L within 12 h postoperatively, ^21^ >2 mmoL/L 30 min after surgery,^22^ >3 mmoL/L 24 h after surgery^23^ and >4 mmoL/L 6 h postoperatively have shown to be predictive values.^24^

Lactate is a biochemical element that is elevated in acute inflammatory pictures with different etiologies and indicates worse outcomes.^5^ In intracranial tumor surgeries, the lactate level >2 mmoL/L was not related to mortality, but it was a predictor of longer duration of the surgical procedure and longer hospital length of stay.^25^ However, to date, studies addressing its measurement in patients with HNSCC undergoing surgical treatment are scarce. The Egyptian study evaluated the predictive value of serial lactate measurement in head and neck cancer,^6^ identifying significantly higher values ​​in the group that did not survive; however, it did not establish a cutoff point that would be a predictor of complications.

In our study, it was possible to identify that an arterial lactate value of 1.7 mmoL/L was the cutoff point with the best discrimination power (*p* =  0.03). The sample size and the fact that it is a single-center study are considered the main study limitations, which hinders comparisons.

## Conclusion

The study showed that arterial lactate is a good predictor of postoperative complications in the surgical treatment of HNSCC. The importance of this finding is the fact that lactate is easily collected and is a low-cost analysis, being a parameter that is routinely requested for surgical patients. Moreover, its accuracy, when performed on the first postoperative day, may include it as one of the criteria to define hospital discharge in this group of patients.

## Conflicts of interest

The authors declare no conflicts of interest.
